# The Traditional Japanese Herbal Medicine Hachimijiogan Elicits Neurite Outgrowth Effects in PC12 Cells and Improves Cognitive in AD Model Rats via Phosphorylation of CREB

**DOI:** 10.3389/fphar.2017.00850

**Published:** 2017-11-21

**Authors:** Kaori Kubota, Haruka Fukue, Hitomi Sato, Kana Hashimoto, Aya Fujikane, Hiroshi Moriyama, Takuya Watanabe, Shutaro Katsurabayashi, Mosaburo Kainuma, Katsunori Iwasaki

**Affiliations:** ^1^Department of Neuropharmacology, Faculty of Pharmaceutical Sciences, Fukuoka University, Fukuoka, Japan; ^2^Institute for Aging and Brain Sciences, Fukuoka University, Fukuoka, Japan; ^3^Community Medicine Education Unit, Department of Clinical Medicine, Faculty of Medical Sciences, Kyushu University, Fukuoka, Japan

**Keywords:** Alzheimer’s disease, Hachimijiogan, neurotrophic factors, CREB, PC12 cells

## Abstract

Hachimijiogan (HJG) is a traditional herbal medicine that improves anxiety disorders in patients with dementia. In this study, we tested the hypothesis that HJG exerts neurotrophic factor-like effects to ameliorate memory impairment in Alzheimer disease (AD) model rats. First, we describe that HJG acts to induce neurite outgrowth in PC12 cells (a rat pheochromocytoma cell line) like nerve growth factor (NGF) in a concentration-dependent manner (3 μg/ml HJG, *p* < 0.05; 10–500 μg/ml HJG, *p* < 0.001). While six herbal constituents of HJG, Rehmannia root, Dioscorea rhizome, Rhizoma Alismatis, Poria sclerotium, Moutan bark, and Cinnamon bark, could induce neurite outgrowth effects, the effect was strongest with HJG (500 μg/ml). Second, we demonstrated that HJG-induced neurite outgrowth was blocked by an inhibitor of cAMP response element binding protein (CREB), KG-501 (10 μM, *p* < 0.001). Moreover, HJG was observed to induce CREB phosphorylation 20–90 min after treatment (20 min, 2.50 ± 0.58-fold) and CRE-mediated transcription in cultured PC12 cells (500 μg/ml, *p* < 0.01; 1000 μg/ml, *p* < 0.001). These results suggest a CREB-dependent mechanism underlies the neurotrophic effects of HJG. Finally, we examined improvements of memory impairment following HJG treatment using a Morris water maze in AD model animals (CI + Aβ rats). Repeated oral administration of HJG improved memory impairment (300 mg/kg, *p* < 0.05; 1000 mg/kg, *p* < 0.001) and induced CREB phosphorylation within the hippocampus (1000 mg/kg, *p* < 0.01). Together, our results suggest that HJG possesses neurotrophic effects similar to those of NGF, and can ameliorate cognitive dysfunction in a rat dementia model via CREB activation. Thus, HJG could potentially be a substitute for neurotrophic factors as a treatment for dementia.

## Introduction

Recently, there have been global increases in the number of patients with dementia disorders, such as AD. However, as the pathogenesis of dementia has not yet been fully elucidated, radical curative therapies have not been established. Novel dementia drugs that do not adversely affect ADL are required. In the study of neurodegenerative diseases characterized by neuronal cell death (such as AD), the role of neurotrophic factors has recently drawn attention. Neurotrophic factors are substances that promote neuronal survival, differentiation, and regeneration. Although various trophic factors have been identified, research has indicated particularly important roles for NGF and BDNF (Obara and Nakahata, 2002).

Nerve growth factor is abundant throughout the hippocampus and cerebral cortex, where it stimulates the production, transportation, and secretion of acetylcholine (Ayer-LeLievre et al., 1988; Jonhagen, 2000). Cholinergic neurons, which are NGF susceptible and play an important role in learning and memory, have been shown to be markedly degenerated in the brain of patients with AD. NGF can modulate the metabolism of amyloid precursor protein from amyloidogenic toward non-amyloidogenic processing via binding to the TrkA (Canu et al., 2017). These features guide the design of therapeutic for AD intending to preserve cholinergic function and anti-amyloidogenic activity. However, neurotrophic factors are macromolecular proteins, with an *in vivo* half-life that is too short to enable delivery to a target tissue or cell. This makes it difficult to directly apply neurotrophic factors as therapeutic agents for AD (Pollack and Harper, 2002; Skaper, 2008). Therefore, attention has turned to developing compounds that show neurotrophic factor-like effects by activating intracellular neurotrophic factor signal transduction pathways and/or promoting biosynthesis of neurotrophic factors. For example, we demonstrated that the Japanese herbal medicine Yokukansan can improve symptoms of dementia (Nogami et al., 2013; Uchida et al., 2013), and its action involves neurotrophic factors such as NGF (Kubota et al., 2013).

In recent years, an association has been reported between neuronal death in AD and decreased activity of CREB (Yamamoto-Sasaki et al., 1999). CREB is a downstream transcriptional regulator of intracellular signaling by neurotrophic factors. CREB is activated through the following three pathways: (1) ERK 1/2; (2) PI3K/Akt; and (3) phospholipase C-γ, all of which lead to CREB phosphorylation (Kaplan and Miller, 2000). In addition to these pathways, AC and the cAMP signaling pathway are also involved in activating CREB and CREB-regulated gene transcription. Activated CREB regulates the transcription of various genes, subsequently inducing spine morphological changes such as neurite outgrowth and branching, and producing long-term potentiation. Additionally, the involvement of CREB in the formation of short-term memories via BDNF induction has been reported (Lonze and Ginty, 2002; Frankland and Bontempi, 2005). Thus, CREB and neurotrophic factor signaling pathways are deeply involved in the formation of both long-term and short-term memory, and are potential targets for drugs to improve cognitive function.

Hachimijiogan (Ba-Wei-Di-Huang-Wan) is an herbal medicine used in China and Japan comprising eight herbs: RR, Cornus fruit, DR, AR, PS, MB, CB, and Aconite root. HJG has traditionally been used to treat diabetes mellitus, hypertension, and nephrotic syndrome. It has also been used to treat dysuria, lumbago, lack of energy, and poor eyesight in older individuals. Furthermore, it has been reported that HJG treatment improved cognitive dysfunction in patients with dementia (Iwasaki et al., 2004). Additionally, several studies, including one of our own, have reported that HJG improves cognitive dysfunction in model animals (Hirokawa et al., 1994, 1996; Moriyama et al., 2017). However, further research is needed to confirm the effects of HJG on cognitive dysfunction. Therefore, to investigate the appropriate clinical application of HJG, we investigated the effects of HJG and its constituent herbs on CREB and neurotrophic factor signaling pathways.

## Materials and Methods

### Materials

Dry powdered extracts of HJG and its constituents (excluding Cornus fruit and Aconite root) supplied by Tsumura & Co. (Tokyo, Japan) were dissolved in distilled water. HJG is a dried extract of the following eight herbs: RR (6.0 g, the root of *Rehmannia glutinosa* Libosch var. purpurea Makino, Scrophulariaceae), Cornus fruit (3.0 g, the fruit of *Cornus officinalis* Siebold et Zucc, Cornaceae), DR (3.0 g, the rhizome of *Dioscorea batatas* Decne, Dioscoreaceae), AR (3.0 g, the rhizome of *Alisma orientale* Juzepczuk, Alismataceae), PS (3.0 g, the sclerotium of *Poria cocos* Wolf, Polyporaceae), MB (2.5 g, the root bark of *Paeonia suffruticosa* Andrews, Paeoniaceae), CB (1.0 g, the bark of *Cinnamomum cassia* Blume, Lauraceae), and Aconite root (0.5 g, the root of *Aconitum carmichaelii* Debeaux, Ranunculaceae).

Nerve growth factor 2.5S was obtained from Life Technologies (Carlsbad, CA, United States). Dimethyl sulfoxide and KG-501 (a CREB inhibitor) were purchased from Sigma–Aldrich (St. Louis, MO, United States). Anti-CREB, anti-phospho-CREB, and anti-rabbit horseradish peroxidase-conjugated secondary antibodies were purchased from Cell Signaling Technology (Beverly, MA, United States). Anti-rabbit β-actin antibodies were obtained from Abcam (Cambridge, United Kingdom).

Donepezil hydrochloride was purchased from Tokyo Chemical Industry (Tokyo, Japan). For animal treatments, HJG and DPZ were dissolved in distilled water. Three weeks after CI, HJG, DPZ, and vehicle (distilled water) were orally administered daily for 7 days.

### Cell Culture

PC12 (rat pheochromocytoma) cells were cultured in RPMI-1640 with 5% (v/v) fetal bovine serum, 10% (v/v) horse serum, 100 units/ml penicillin, and 100 μg/ml streptomycin. Cells were grown to confluence at 37°C in 5% CO_2_. The medium was changed two or three times per week. Cells treated with NGF (50 ng/ml) were used as positive controls.

### Neurite Outgrowth Assay

PC12 cells (1 × 10^5^ cells/ml) were seeded on collagen-coated six-well plates and cultured for 24 h. Cells were treated with different dilutions of HJG (1–500 μg/ml), or each herb at the concentration based on the original mixing ratio of constituents, and cultured for 72 h. PC12 cells were photographed using an inverted microscope (Olympus, Tokyo, Japan) and phase-contrast objectives. Images of two fields per well were taken with 40–50 cells per field. The length and number of neurites were measured for 20 independent cells per field using Image J 1.44 software (NIH Image Software), and the neurite lengths for each cell were summed. Each experiment was conducted in triplicate.

### Western Blotting

Proteins were extracted from cells at appropriate time points after drug treatment using RIPA buffer [50 mM Tris–HCl (pH 8), 150 mM NaCl, 1% NP40, 0.5% sodium deoxycholate, 0.1% SDS] containing a protease inhibitor cocktail (Nacalai Tesque, Kyoto, Japan). Protein concentrations were measured using a BCA Protein Assay Reagent Kit (Thermo Fisher Scientific, Waltham, MA, United States). Proteins were separated on SDS polyacrylamide gels, transferred to polyvinylidene fluoride membranes, and probed with specific antibodies. Immunoreactive polypeptides were detected with chemiluminescence using ImmunoStar LD (Wako, Osaka, Japan).

### Luciferase Reporter Assay

PC12 cells (1 × 10^5^ cells/well) were seeded in collagen-coated 12-well plates for 24 h. Cells were co-transfected with the pGL4.29 luciferase reporter vector containing a CRE and pGL4.74 Renilla luciferase vector (Promega) using Lipofectamine 3000 (Life Technologies). Forty-eight hours after transfection, cells were treated with vehicle (0.1% DMSO), HJG, or its constituents for 8 h and harvested using Passive Lysis Buffer (Promega).

Luciferase activities were determined with a Dual-Luciferase Reporter Assay System Kit (Promega) according to the manufacturer’s instructions. We normalized the intensity of luciferase reactions measured in the lysates of transfectants to their Renilla luciferase activity, which was used as an internal control.

### Animals

Male Wistar rats weighing 250–300 g were obtained from Kyudo Co., Ltd. (Saga, Japan). Rats were housed in groups of 4–5 per cage at 23 ± 2°C with a relative humidity of 60 ± 10%, and maintained under a 12-h light–dark cycle. Food and water were available *ad libitum* except during the restricted feeding period. We restricted food intake for rats (8–10 g each day), and their body weight was maintained at ∼80% of free-feeding levels during the eight-arm radial maze tasks. We conducted all procedures relating to animal care and use according to regulations dictated by the Experimental Animal Care and Use Committee of Fukuoka University (No. 1405744).

### Preparation of Aggregated Aβ and CI + Aβ Rats

Aggregated Aβ was prepared as previously described (Moriyama et al., 2017). Aβ42 peptides were purchased from AnaSpec Inc. (Fremont, CA, United States), dissolved in HEPES-buffered solution to a final concentration of 10 μM, and incubated at 37°C for 7 days.

Cerebral ischemia + Aβ rats were prepared by combining an intracerebroventricular Aβ injection with transient CI as previously described (Moriyama et al., 2017). Rats were anesthetized with sodium pentobarbital. After exposure and threading of their bilateral common carotid arteries, rats were placed in a stereotaxic frame. The bilateral vertebral arteries were electrocauterized with a bipolar coagulator (MICRO-3D; Mizuho Industrial, Tokyo, Japan). For intracerebroventricular infusion, a guide cannula (0.71 ± 0.02 mm o.d., 0.41 ± 0.02 mm i.d., 13-mm length) was implanted bilaterally into the lateral cerebral ventricles at AP = -0.8 mm, *L* = ±1.3 mm, and *H* = 3.3 mm from the bregma. Three days after cannulation, the common carotid arteries were compressed by clips and cerebral circulation was interrupted for 10 min to create CI.

The first injection of Aβ was performed 1 h after CI. Aβ was injected daily for 7 days. Aβ (600 pmol/20 μl/day) was injected bilaterally using injection cannulas (0.35 ± 0.01 mm o.d., 0.17 ± 0.02 mm i.d., 14-mm length) connected by polyethylene tubing to a perfusion pump (CMA/100; Microdialysis AB, Stockholm, Sweden) and driven at a rate of 1 μl/min.

### Morris Water Maze

Apparatus and procedures for the water maze (swimming pool from Neuroscience Inc., Tokyo, Japan; 150-cm diameter, 45-cm depth) were modified from those originally developed by Morris (1981) and essentially identical to those described previously (Ikeda et al., 2001). The pool was filled with clear water at 23 ± 2°C. Each rat underwent three trials daily for 5 consecutive days.

A trial consisted of placing the rat by hand into the water facing the wall of the pool at one of three starting positions located in a quadrant that did not contain the platform. The platform (12 cm in diameter) was located in a constant position in the middle of one quadrant with its top surface located 2 cm below the surface of the water. During each block of three trials, the rat started once at each of three starting positions. Rats that demonstrated a swimming time on the 5th day within 20 s of the average were deemed to have memorized the maze. In the probe test, the platform was removed from the tank and rats were allowed to swim freely for 120 s. Their performance in the probe test was assessed by swimming time to first reach the platform position.

### Statistical Analyses

Results are indicated as mean ± SEM for three or more experiments. Experiments with two groups were analyzed using Student’s *t*-test. Differences between the control group and each treatment group were subjected to one-way analysis of variance (ANOVA) followed by Dunnett’s test. Multiple comparisons were evaluated with Tukey’s test after one-way ANOVA. Values of *p* < 0.05 were considered statistically significant.

## Results

### Effects of HJG on Neurite Outgrowth in PC12 Cells

**FIGURE 1:**
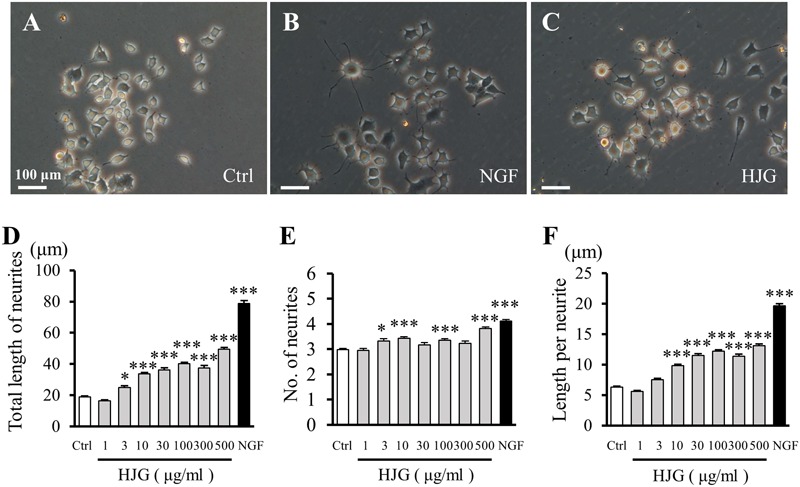
Effects of HJG on neurite outgrowth of PC12 cells. PC12 cells were cultured for 72 h in normal medium **(A)**, medium with 50 ng/ml NGF **(B)**, or 500 μg/ml HJG **(C)**, and morphological appearance was then observed. Scale bar = 100 μm. Total length of neurites **(D)**, numbers of neurites **(E)**, and length per neurite **(F)** were analyzed as described in the Section “Materials and Methods”. Data represent mean ± SEM from three independent experiments. ^∗^*p* < 0.05 and ^∗∗∗^*p* < 0.001 represent significant differences compared with control (ANOVA followed by Dunnett’s test).

Derived from rat pheochromocytomas, PC12 cells are an *in vitro* neuronal model characterized by the extension of protrusions and differentiation into sympathetic cells following stimulation with NGF (**Figures 1A,B**). PC12 cells treated with HJG for 3 days exhibited clearly different morphologies compared with the control group, including protrusion elongation (**Figure 1C**). By measuring the length and number of neurites, we observed a significant increase in total neurite length as the concentration of HJG increased from 3 to 500 μg/ml compared with the control group (**Figure 1D** and Supplementary Table S1). Furthermore, an increased number of neurites were observed following treatment with 3–500 μg/ml HJG compared with the control group (**Figure 1E** and Supplementary Table S1). We also calculated the mean length per neurite by dividing the total length of measured neurites by the number of neurites. This showed that the length per neurite increased significantly compared with the control group as the concentration of HJG increased from 10 to 500 μg/ml (**Figure 1F** and Supplementary Table S1). Thus, HJG induces neurite outgrowth in a concentration-dependent manner and, furthermore, this effect was mediated by an increase in the lengths of individual neurites, rather than an increase in the number of neurites.

### Effects of HJG Constituents on Neurite Outgrowth of PC12 Cells

**FIGURE 2:**
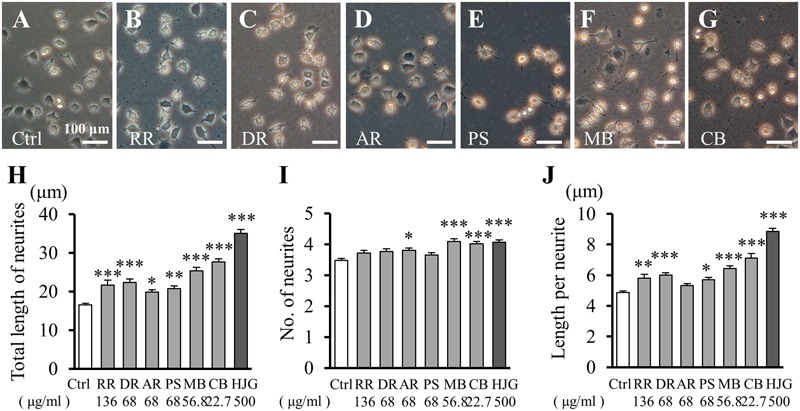
Effects of HJG constituents on neurite outgrowth of PC12 cells. PC12 cells were incubated for 72 h with each of the following constituent herbs: **(A)** Ctrl, **(B)** RR, 136 μg/ml, **(C)** DR, 68 μg/ml, **(D)** AR, 68 μg/ml, **(E)** PS, 68 μg/ml, **(F)** MB, 56.8 μg/ml, **(G)** CB, 22.7 μg/ml, and HJG, 500 μg/ml. Scale bar = 100 μm. Total length of neurites **(H)**, numbers of neurites **(I)**, and length of individual neurites **(J)** were observed. Data represent mean ± SEM from three independent experiments. ^∗^*p* < 0.05, ^∗∗^*p* < 0.01, and ^∗∗∗^*p* < 0.001 represent significant differences compared with controls (ANOVA followed by Dunnett’s test).

The above results suggested that HJG exerts neurite outgrowth effects similar to NGF. To identify the effects of each active constituent of HJG, we next treated cells with its six herbal constituents and observed neurite outgrowth (**Figures 2A–G**). Neurite outgrowth was represented by neurite extension (**Figure 2H**), neurite numbers (**Figure 2I**), and length per individual neurite (**Figure 2J**). Using these methods, we searched for effective herbal constituents of HJG capable of exerting an elongation effect. The total length of neurites was increased significantly compared with the control group (**Figure 2H** and Supplementary Table S2). These results suggest each six herbal constituents has a neurotrophic factor-like neurite outgrowth effect.

The number of neurites increased significantly compared with controls following treatment with AR, MB, and CB (**Figure 2I** and Supplementary Table S2). Notably, the lengths of individual neurites also increased significantly compared with the control group following treatment with five of the constituents, but not AR (**Figure 2J** and Supplementary Table S2).

Therefore, all of the herbal constituents of HJG showed a neurite outgrowth effect, although their effects differed slightly depending on the particular constituent tested. Furthermore, these effects were mediated by an increase in the length of individual neurites rather than the total number of neurites.

### Effect of HJG on CREB Downstream of Intracellular Neurotrophic Factor Signaling Pathways

**FIGURE 3:**
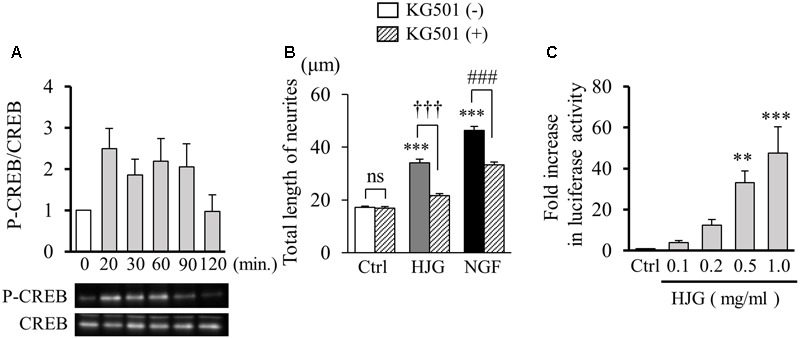
Effect of HJG on CREB activation in PC12 cells. **(A)** HJG induces CREB phosphorylation in a time-dependent manner. PC12 cells were untreated (0 min) or treated with 0.5 mg/ml HJG for 20–120 min. **(B)** KG-501 inhibited HJG-induced neurite outgrowth of PC12 cells. PC12 cells were pretreated with KG-501 (10 μM) for 30 min and incubated for 3 days in media containing HJG (0.5 mg/ml) or NGF (50 ng/ml) before assessing the total length of neurites. Data represent mean ± SEM from three independent experiments. ^∗∗∗^*p* < 0.001 indicates significant differences compared with untreated controls (ANOVA followed by Tukey’s test). ^†††^*p* < 0.001 indicates significant difference compared with KG-501 + HJG-treated cells and HJG-treated cells (Student’s *t*-test). ^###^*p* < 0.001 indicates significant difference compared with KG-501 + NGF-treated cells and NGF-treated cells (Student’s *t*-test). **(C)** PC12 cells were transfected with a CRE-mediated luciferase reporter construct and Renilla luciferase control plasmid for 48 h, and cells were subsequently untreated (Ctrl) or treated with 0.1, 0.2, 0.5, or 1.0 mg/ml HJG for 8 h. Luciferase activities were measured in lysates of transfectants and normalized to their Renilla luciferase control activity. Data represent mean ± SEM from three independent experiments. ^∗∗^*p* < 0.01 and ^∗∗∗^*p* < 0.001 represent significant differences compared with controls (ANOVA followed by Dunnett’s test).

We investigated the effect of HJG on CREB, which exists downstream of neurotrophic factor intracellular signaling pathways. CREB plays an important role in neurogenesis and memory formation (**Figure 3**). Examination of CREB phosphorylation with Western blots showed increased phosphorylation of CREB 20–90 min after treatment with HJG, suggesting CREB activation by HJG (0 min, 1.00 ± 0.00-fold, *n* = 6; 20 min, 2.50 ± 0.58-fold, *n* = 6; 30 min, 1.86 ± 0.45-fold, *n* = 6; 60 min, 2.19 ± 0.65-fold, *n* = 6; 90 min, 2.05 ± 0.66-fold, *n* = 6; 120 min, 0.97 ± 047-fold, *n* = 6, **Figure 3A**). Subsequently, we investigated neurite outgrowth induced by HJG administered with KG-501 (10 μM), a CREB inhibitor. Co-treatment with KG-501 significantly inhibited the neurite outgrowth effect of HJG (Ctrl, 17.24 ± 0.49 μm, *n* = 360; KG-501, 16.92 ± 0.57 μm, *n* = 360, ns vs. Ctrl; HJG, 34.14 ± 1.26 μm, *n* = 360, *p* < 0.001 vs. Ctrl; HJG+KG-501, 21.65 ± 0.82 μm, *n* = 360, *p* < 0.001 vs. HJG; NGF, 46.37 ± 1.55 μm, *n* = 360, *p* < 0.001 vs. Ctrl; NGF+KG-501, 33.32 ± 1.13 μm, *n* = 360, *p* < 0.001 vs. NGF, **Figure 3B**).

Cyclic AMP response element-binding protein, a transcription factor, is known to increase the expression of target genes in a CRE-dependent manner. Therefore, we examined the effect of HJG on transcriptional activity of CREB using a CRE reporter vector. As a result, we confirmed a significant and concentration-dependent increase in CRE-dependent transcriptional activity with HJG treatment (Ctrl, 1.00 ± 0.003-fold, *n* = 8; HJG, 0.1 mg/ml, 4.07 ± 1.13-fold, *n* = 6; HJG, 0.2 mg/ml, 12.51 ± 3.22-fold, *n* = 6; HJG, 0.5 mg/ml, 33.45 ± 6.82-fold, *n* = 6, *p* < 0.01 vs. Ctrl; HJG, 1.0 mg/ml, 48.83 ± 15.18-fold, *n* = 6, *p* < 0.001 vs. Ctrl, **Figure 3C**).

These results demonstrate repression of the neurite outgrowth effect of HJG by a CREB inhibitor, while HJG treatment leads to CREB phosphorylation and increased CRE-dependent transcriptional activity. Together, these results show that HJG functions by activating CREB.

### HJG Constituents Contribute to Neurite Outgrowth via CREB

**FIGURE 4:**
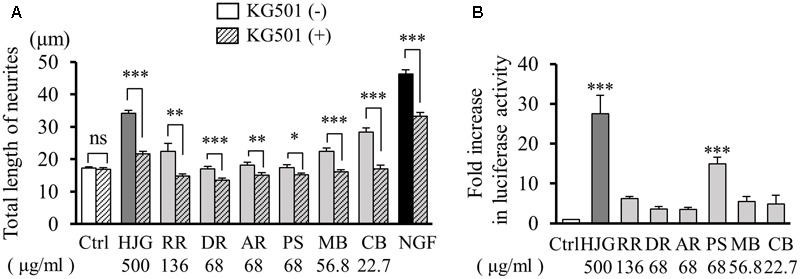
Effects of HJG constituents on CREB activation in PC12 cells. **(A)** KG-501 inhibited HJG constituent-induced neurite outgrowth in PC12 cells. PC12 cells were pretreated with KG-501 (10 μM) for 30 min and incubated for 3 days in media containing HJG constituents or NGF before assessing the total length of neurites. Data represent mean ± SEM from three independent experiments. ^∗^*p* < 0.05, ^∗∗^*p* < 0.01, and ^∗∗∗^*p* < 0.001 indicate significant differences between KG-501 and each HJG constituent treated cells and no-treated KG-501 constituent treated cells (Student’s *t*-test). **(B)** PC12 cells were transfected with a CRE-mediated luciferase reporter construct and Renilla luciferase control plasmid for 48 h, and cells were subsequently treated with HJG constituents for 8 h or left untreated as a control. Luciferase activities were measured in lysates of transfectants and normalized to their Renilla luciferase control activity. Data represent mean ± SEM from three independent experiments. ^∗∗∗^*p* < 0.001 represents significant differences compared with controls (ANOVA followed by Dunnett’s test).

Thus far, our results confirm that HJG induces neurotrophic factor-like neurite outgrowth, and CREB plays an important role in this effect. To clarify which HJG constituent was important for this CREB-mediated neurotrophic factor-like effect, we investigated neurite outgrowth following treatment of rats with HJG constituents and KG-501 (10 μM), a CREB inhibitor. Neurite outgrowth was observed following treatment with both HJG and its six constituents, and was suppressed by KG-501 treatment (**Figure 4A** and Supplementary Table S3).

Next, we examined the effect of each constituent on CREB transcriptional activity using a CRE reporter vector. Among the HJG constituents, we observed particularly significant increases of CRE-dependent transcription activity following treatment with PS (**Figure 4B**). However, while PS showed a particularly strong effect, CREB-dependent neurite outgrowth was also observed following treatment with the other five herbal constituents (Ctrl, 1.00 ± 0.005-fold, *n* = 5; HJG, 27.48 ± 4.63-fold, *n* = 4, *p* < 0.001 vs. Ctrl; RR, 6.19 ± 0.59-fold, *n* = 4; DR, 3.54 ± 0.65-fold, *n* = 4; AR, 3.54 ± 0.47-fold, *n* = 4; PS, 14.90 ± 1.65-fold, *n* = 4, *p* < 0.001 vs. Ctrl; MB, 5.50 ± 1.22-fold, *n* = 4; CB, 4.87 ± 2.22-fold, *n* = 4, **Figure 4B**). Furthermore, as there exist various pathways by which neurite outgrowth occurs, we acknowledge that factors and pathways other than CREB likely contribute to the effect of HJG on neurite outgrowth.

### HJG Improves Memory Impairments by Activating CREB in the Hippocampus of Dementia Model Rats

**FIGURE 5:**
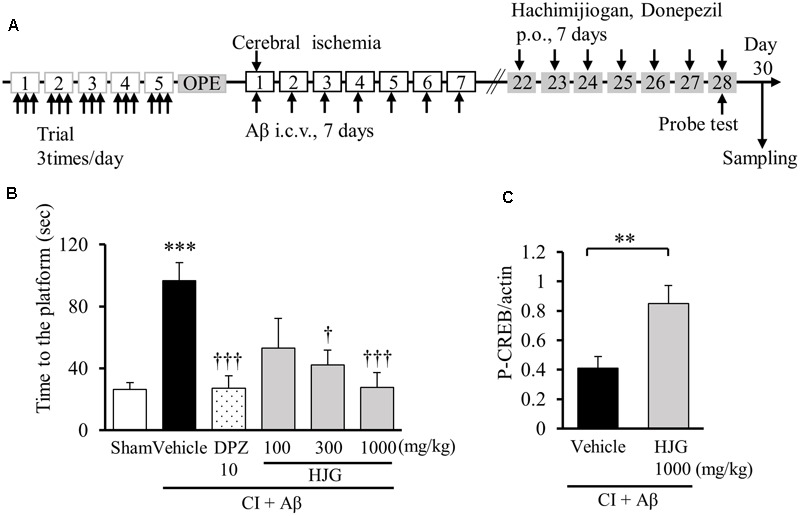
Effect of HJG on memory impairment in rats treated with CI and Aβ protein (CI + Aβ). **(A)** Schedule of experiment. Each rat underwent three trials daily for 5 consecutive days for memory acquisition. CI + Aβ rats were prepared by a combining intracerebroventricular Aβ injection with transient CI. On days 22–28 after CI, HJG, DPZ, or vehicle (water) was administered daily for 7 days. On day 28, cognitive function was analyzed with the probe test. On day 30, samples were collected from rat brains for Western blotting. **(B)** Swimming time to first reach the platform position. The probe test was performed 1 h after the 7th HJG treatment. Rats were allowed to swim freely for 180 s. Data represent mean ± SEM. ^∗∗∗^*p* < 0.001 represents significant differences compared with Sham (Student’s *t*-test). ^†^*p* < 0.05 and ^†††^*p* < 0.001 indicate significant differences between Vehicle (one-way ANOVA followed by Dunnett’s test). **(C)** HJG induces CREB phosphorylation in the hippocampus of CI + Aβ rats. Data represent mean ± SEM. ^∗∗^*p* < 0.01 indicates significant differences compared with vehicle (Student’s *t*-test).

After confirming that HJG has neurotrophic factor-like effects and activates CREB *in vitro*, we subsequently used an animal model of dementia to examine whether HJG ameliorates memory disorder using the Morris water maze (**Figure 5A**). In animals with combined CI and intraventricular administration of Aβ (CI + Aβ rats), treatment with HJG showed a concentration-dependent amelioration (i.e., shortening) of previously significantly longer swim times to first reach the platform position (Sham, 26.41 ± 4.41 s, *n* = 16; Vehicle, 96.61 ± 11.72 s, *n* = 17, *p* < 0.001 vs. Sham; HJG, 100 mg/kg, 53.02 ± 19.21 s, *n* = 7; HJG, 300 mg/kg, 42.11 ± 9.75 s, *n* = 7, *p* < 0.05 vs. Vehicle; HJG, 1000 mg/kg, 27.76 ± 9.53 s, *n* = 9, *p* < 0.001 vs. Vehicle; **Figure 5B**). DPZ, an inhibitor of acetylcholinesterase, also reduced swim time (DPZ, 27.24 ± 7.83 s, *n* = 13, *p* < 0.001 vs. Vehicle). There were no significant differences in swimming speed between any of the groups (data not shown). Therefore, both HJG and DPZ (a treatment for AD) showed similar amelioration of memory disorder in dementia model rats.

Given the confirmation that HJG improved cognitive function, we subsequently examined activation of CREB by HJG in the brain of CI + Aβ rats. Phosphorylated CREB protein in the hippocampus was quantified with Western blot (**Figure 5C**). When dementia model rats underwent 7 days of continuous oral HJG administration, there was a significant increase in phosphorylated CREB (Vehicle, 0.41 ± 0.08-fold, *n* = 12; HJG, 1000 mg/kg, 0.85 ± 0.12-fold, *n* = 8, *p* < 0.01 vs. Vehicle). Therefore, we suggest that HJG improved memory impairment by restoring phosphorylated CREB in the hippocampus of CI + Aβ rats.

## Discussion

Previously, HJG has been used to treat the symptoms of dysuria and benign prostatic hyperplasia in elderly patients. However, in recent years it has been reported that HJG also improves cognitive function (Hirokawa et al., 1994, 1996; Iwasaki et al., 2004; Moriyama et al., 2017). Therefore, we believed it was important to clarify the mechanisms by which HJG improved cognitive function, as this could enable an extension of the therapeutic uses of HJG. From our previous research, we reported that Yokukansan, a Japanese herbal medicine, has neurotrophic factor-like effects (Kubota et al., 2013) and can improve the symptoms of dementia (Nogami et al., 2013; Uchida et al., 2013). Therefore, we hypothesized that neurotrophic factors and their signaling pathways may be associated with improved cognitive function following HJG treatment.

First, we examined neurite outgrowth following HJG treatment by examining neurite elongation effects in PC12 cells. Our results showed that HJG induced neurite outgrowth in a similar manner to neurotrophic factors. In addition, our results suggested that the constituents of HJG did not significantly influence the total number of neurites, but did elicit neurite elongation activity. We next attempted to identify the candidate constituent herb(s). As shown in **Figure 2**, all eight constituent herbs showed neurite elongation actions, although each was weaker than that of HJG. These results suggest that the neurite elongation activity of HJG is not caused by a particular herb, but by the additive and/or synergistic effects of each herb. These results also suggest that many compounds, at least eight, are involved in the neurite elongation action of HJG. At present, it is difficult to identify all of these active compounds. Furthermore, we confirmed that CREB present downstream of the neurotrophic factor signaling system, mediates the neurite outgrowth actions of HJG and each constituent herb (**Figures 3, 4**). In the current study, we showed that the CREB-mediated pathway is mechanistically related to the neurotropic effects of HJG. Considering that HJG is a multicomponent drug, other mechanisms may also be associated with these effects, which is an important point for understanding the complete neuropharmacological actions of HJG.

We previously showed that memory impairment in CI + Aβ rats was ameliorated by HJG administration for 7 days after ischemia treatment (Moriyama et al., 2017). In our previous study, we examined the effect of HJG in the acute phase of model animal pathology. In this study, we evaluated improved cognitive function following HJG treatment after the disease condition had been established (4 weeks after CI treatment). After 4 weeks of CI + Aβ treatment, animals showed cognitive dysfunction, which was improved by HJG administration. Improved cognitive function following HJG treatment was observed even when administration occurred at the onset of the disease condition, which more closely resembles clinical conditions.

In this study, HJG elicited CREB phosphorylation in cultured PC12 cells and AD model rats. However, it is still not clear how HJG induces CREB activation in detail. Some candidate pathways are involved in activation of CREB. Many neurotrophic factors and neurotransmitters act as membrane receptors to trigger signaling pathways that regulate CREB phosphorylation (Carlezon et al., 2005). Phosphodiesterase (PDE), which is part of the CREB regulation pathway, is drawing attention as a target for treatment of psychiatric and neurodegenerative disorders (Menniti et al., 2006; Medina, 2011). PDE4 inhibitor phosphorylates CREB protein, enhances the induction of long-term potentiation within the hippocampus, and is involved in the retention of long-term memory (McGirr et al., 2016). It is also known that PDE inhibitors are effective for treating depression (O’Donnell and Zhang, 2004). It was suggested that the antidepressant effects of PDE inhibition result from upregulation of BDNF (Monnet, 2005) and the promotion of neurogenesis within the hippocampus (Nakagawa et al., 2002). Furthermore, various alkaloids, flavonoids, and saponins from plants have demonstrated PDE inhibitory activity and CREB phosphorylation activity without activating neurotrophic receptors (Rahimi et al., 2010). Notably, in our preliminary experiments, HJG increased intracellular cAMP concentration. Thus, we hypothesize that candidate compounds in HJG might exert PDE inhibitor activity and stimulate the CREB pathway through increased cAMP levels.

Cyclic AMP response element-binding protein has been reported to regulate the transcription of various genes, as well as neuronal differentiation, cell survival, protrusion elongation, and memory/learning ability (Lonze and Ginty, 2002). The activation of CREB by phosphorylation occurs following interaction with the transcriptional activator CREB protein, which initiates transcription and translation of CREB target genes to induce the synaptic plasticity necessary for long-term memory formation. Furthermore, CREB has been shown to promote short-term memory by up-regulating BDNF. Thus, CREB signaling appears to be involved in both short-term and long-term memory (Suzuki et al., 2011). Genes regulated by CREB include CREB itself, neurotrophic factors such as BDNF, TrkB, fibroblast growth factor 6, insulin-like growth factor 1, neuropeptides and their receptors, and cell death regulatory molecules such as Bcl-2. It has also been reported that accumulation of Aβ may lead to reduced CREB phosphorylation (Vitolo et al., 2002), while CREB-induced gene expression is impaired in both AD model mice and the brains of patients with AD (Phillips et al., 1991; Gong et al., 2004). Therefore, increasing this neurotrophic factor and activating this pathway offer a potential AD treatment.

## Conclusion

We have shown that HJG exerts neurotrophic effects via CREB activation, similar to that of neurotrophic factors. Furthermore, HJG ameliorates cognitive dysfunction in dementia model rats via CREB activation. Our results suggest that HJG could be a potential substitute for neurotrophic factors as a treatment for dementia. As CREB is not only related to memory formation but also depression and anxiety (Carlezon et al., 2005; Manning et al., 2017), HJG may be effective for treating the behavioral and psychological symptoms of dementia, as well as other psychiatric and neurological diseases.

## Author Contributions

KK, TW, and KI designed the study. KK, HF, HS, KH, AF, and HM conducted the research and analyzed the data. KK, TW, and MK wrote the paper. SK, MK, and KI supervised the experiments. All authors read and approved the final manuscript.

## Conflict of Interest Statement

The authors declare that the research was conducted in the absence of any commercial or financial relationships that could be construed as a potential conflict of interest.

## Abbreviations

Aβ: amyloid β-peptide
AC: adenylyl cyclase
AD: Alzheimer’s disease
ADL: activities of daily living
AR: Alismatis rhizome
BDNF: brain-derived neurotrophic factor
CB: Cinnamon bark
CI: cerebral ischemia
CRE: cAMP response element
CREB: cyclic AMP response element-binding protein
DPZ: donepezil hydrochloride
DR: Dioscorea rhizome
ERK: extracellular signal-regulated kinase
HJG: Hachimijiogan
MB: Moutan bark
NGF: nerve growth factor
PI3K: phosphatidylinositol 3-kinase
PS: Poria Sclerotium
RR: rehmannia root
TrkA: tyrosine receptor kinase A
