# Supplementary material for: The Traditional Japanese Herbal Medicine Hachimijiogan Elicits Neurite Outgrowth Effects in PC12 Cells and Improves Cognitive in AD Model Rats via Phosphorylation of CREB
Source: Front Pharmacol. 2017 Nov 21;8:850. doi: 10.3389/fphar.2017.00850 (PMC5702328; doi:10.3389/fphar.2017.00850)
Supplement: Supplementary file 1 [file Table_1.DOCX]

Supplementary Material

The traditional Japanese herbal medicine Hachimijiogan elicits neurite outgrowth effects in PC12 cells and improves cognitive in AD model rats via phosphorylation of CREB

Kaori Kubota*, Haruka Fukue, Hitomi Sato, Kana Hashimoto, Aya Fujikane, Hiroshi Moriyama, Takuya Watanabe, Shutaro Katsurabayashi, Mosaburo Kainuma, Katsunori Iwasaki

*** Correspondence:** Dr. Kaori Kubota: kkubota@fukuoka-u.ac.jp

Supplemental Table 1 (Table S1): Effects of Hachimijiogan (HJG) on neurite outgrowth of PC12 cells.

Supplemental Table 1 (Table S1)


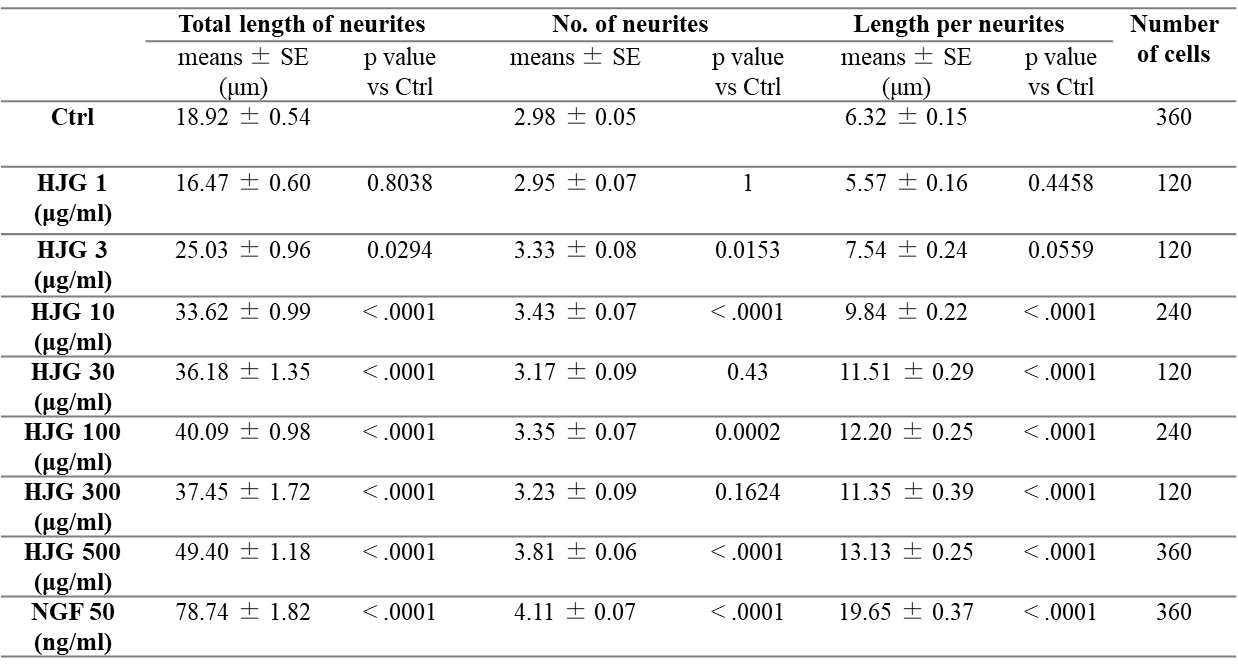


Ctrl, control; HJG, Hachimijiogan; NGF, nerve growth factor. PC12 cells were cultured for 72 h in normal medium (Ctrl), or medium with various concentration of HJG or 50 ng/mL NGF. Total length of neurites, numbers of neurites and length per neurite were analyzed as described in the Materials and Methods. Values are expressed as means ± of three and more experiments. p value between the control group and each treatment group were subjected to one-way analysis of variance (ANOVA) followed by Dunnett’s test. These data are shown as graphs in Fig. 1D (total length of neurites), Fig. 1 E (No. of neurites) and Fig. 1F (length per neurite).
