# Supplementary material for: The Traditional Japanese Herbal Medicine Hachimijiogan Elicits Neurite Outgrowth Effects in PC12 Cells and Improves Cognitive in AD Model Rats via Phosphorylation of CREB
Source: Front Pharmacol. 2017 Nov 21;8:850. doi: 10.3389/fphar.2017.00850 (PMC5702328; doi:10.3389/fphar.2017.00850)
Supplement: Supplementary file 2 [file Table_2.DOCX]

Supplementary Material

The traditional Japanese herbal medicine Hachimijiogan elicits neurite outgrowth effects in PC12 cells and improves cognitive in AD model rats via phosphorylation of CREB

Kaori Kubota*, Haruka Fukue, Hitomi Sato, Kana Hashimoto, Aya Fujikane, Hiroshi Moriyama, Takuya Watanabe, Shutaro Katsurabayashi, Mosaburo Kainuma, Katsunori Iwasaki

*** Correspondence:** Dr. Kaori Kubota: kkubota@fukuoka-u.ac.jp

Supplemental Table 2 (Table S2): Effects of Hachimijiogan (HJG) constituents on neurite outgrowth of PC12 cells.

Supplemental Table 2 (Table S2)


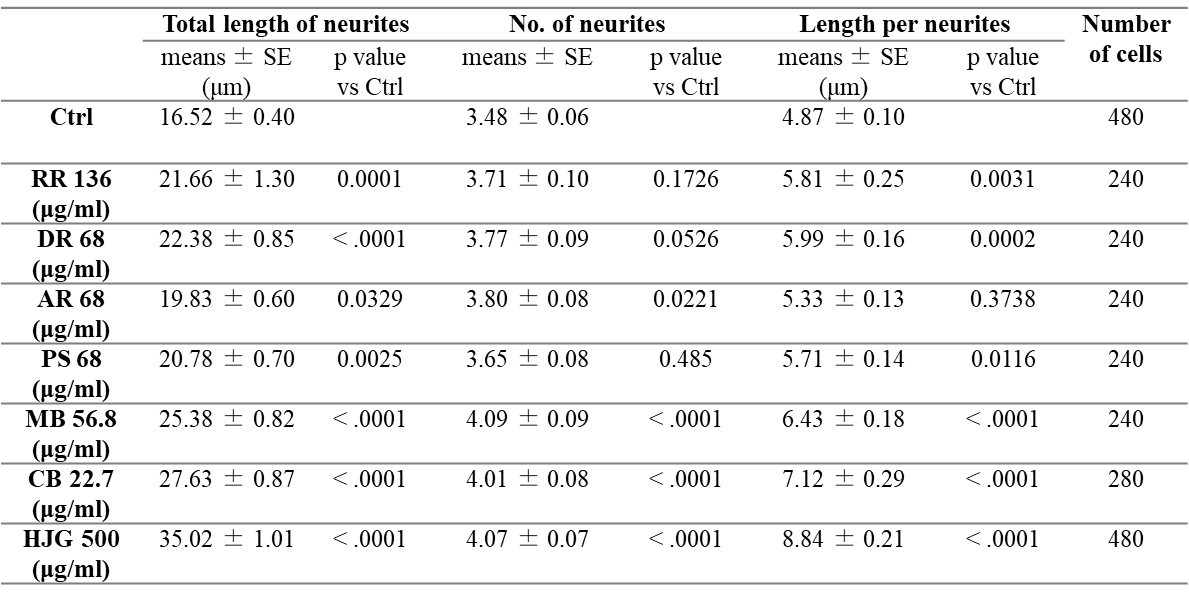


Ctrl, control; RR, Rehmannia root; DR, Dioscorea rhizome; AR, Alismatis Rhizome; PS, Poria sclerotium; MB, Moutan bark; CB, Cinnamon bark; and HJG, Hachimijiogan. PC12 cells were incubated for 72 h with each of the following constituent herbs: RR, 136 μg/ml; DR, 68 μg/ml; AR, 68 μg/ml; PS, 68 μg/ml; MB, 56.8 μg/ml; CB, 22.7 μg/ml and HJG 500 μg/ml. Total length of neurites, numbers of neurites and length of individual neurites were observed. Values are expressed as means ± of three and more experiments. p value between the control group and each treatment group were subjected to one-way analysis of variance (ANOVA) followed by Dunnett’s test. These data are shown as graphs in Fig. 2 H (total length of neurites), Fig. 2 I (No. of neurites) and Fig. 2 J (length per neurite).
